# NMDA Receptor Expression by Retinal Ganglion Cells Is Not Required for Retinofugal Map Formation nor Eye-Specific Segregation in the Mouse

**DOI:** 10.1523/ENEURO.0115-20.2021

**Published:** 2021-07-12

**Authors:** Kristy O. Johnson, Nathan A. Smith, Evan Z. Goldstein, Vittorio Gallo, Jason W. Triplett

**Affiliations:** 1 Center for Neuroscience Research, Children’s National Research Institute, Washington, DC 20010; 2 Institute for Biomedical Sciences, The George Washington University School of Medicine and Health Sciences, Washington, DC 20052; 3 Department of Pediatrics, The George Washington University School of Medicine and Health Sciences, Washington, DC 20052; 4 Department of Pharmacology and Physiology, The George Washington University School of Medicine and Health Sciences, Washington, DC 20052

**Keywords:** activity, development, retinocollicular, retinogeniculate, visual system

## Abstract

Retinal ganglion cells (RGCs) project topographically to the superior colliculus (SC) and dorsal lateral geniculate nucleus (dLGN). Spontaneous activity plays a critical role in retinotopic mapping in both regions; however, the molecular mechanisms underlying activity-dependent refinement remain unclear. Previous pharmacologic studies implicate NMDA receptors (NMDARs) in the establishment of retinotopy. In other brain regions, NMDARs are expressed on both the presynaptic and postsynaptic side of the synapse, and recent work suggests that presynaptic and postsynaptic NMDARs play distinct roles in retinotectal developmental dynamics. To directly test the role of NMDARs expressed by RGCs in retinofugal map formation, we took a conditional genetic knock-out approach to delete the obligate GluN1 subunit of NMDARs in RGCs. Here, we demonstrate reduced GluN1 expression in the retina of Chrnb3-Cre;GluN1^flox/flox^ (pre-cKO) mice without altered expression in the SC. Anatomical tracing experiments revealed no significant changes in termination zone size in the SC and dLGN of pre-cKO mice, suggesting NMDAR function in RGCs is not an absolute requirement for topographic refinement. Further, we observed no change in the eye-specific organization of retinal inputs to the SC nor dLGN. To verify that NMDA induces activity in RGC terminals, we restricted GCaMP5 expression to RGCs and confirmed induction of calcium transients in RGC terminals. Together, these findings demonstrate that NMDARs expressed by RGCs are not required for retinofugal topographic map formation nor eye-specific segregation in the mouse.

## Significance Statement

Topographic organization of retinal inputs in the brain is thought to be critical for the efficient relay of spatial information in the visual scene. Previous studies suggest NMDA receptors (NMDARs) play a crucial role in establishing topography in the superior colliculus; however, these studies could not distinguish between potential presynaptic or postsynaptic roles. Here, we show NMDAR function in retinal ganglion cells (RGCs) is not required for the establishment of topography. Further, we find RGC NMDARs are not required to establish or maintain eye-specific laminae in retinorecipient regions.

## Introduction

Retinal ganglion cells (RGCs) project to two main image forming regions, the superior colliculus (SC) and the dorsal lateral geniculate nucleus (dLGN), where their axon terminals are organized topographically. The establishment of topography occurs in a protracted process during the first week of postnatal life in the mouse (Johnson and Triplett, 2021). Initially, diffuse terminations are refined to topographically appropriate locations in a manner dependent on a combination of molecular cues (Feldheim and O’Leary, 2010), axon-axon competition (Triplett et al., 2011), and neuronal activity (McLaughlin et al., 2003; Pfeiffenberger et al., 2006).

The activity driving retinofugal projection refinement is spontaneous, consisting of highly correlated bursts of action potentials, termed retinal waves, that propagate across the retina (Meister et al., 1991; Wong et al., 1993) and are transferred to downstream areas (Ackman et al., 2012). Retinal waves progress through three stages based on their mode of propagation, mediated first by gap junctions, then acetylcholine, and finally glutamate. Disruption of cholinergic waves perturbs retinotopic map formation in the SC and dLGN (McLaughlin et al., 2003; Chandrasekaran et al., 2007; Cang et al., 2008; Xu et al., 2015). While it is clear the normal pattern of retinal waves is critical for topographic map formation in the SC and dLGN, the molecular mechanisms by which activity mediates these processes remain unclear.

NMDA receptors (NMDARs) are ionotropic glutamate receptors widely expressed throughout the brain and play a critical role in activity-dependent synaptic strengthening (Nicoll and Malenka, 1999). Previous studies suggest a critical role for NMDARs in the establishment of retinocollicular connectivity. Indeed, pharmacological blockade showed a disruption in retinocollicular map organization when applied locally to the SC (Simon et al., 1992) or tectum (Cline and Constantine-Paton, 1989). Further, the receptive field size of SC neurons was increased on chronic blockade, and lesion-induced plasticity was disrupted (Huang and Pallas, 2001). Intriguingly, NMDAR blockade resulted in dramatic changes to retinal arborization dynamics (Rajan et al., 1999; Ruthazer et al., 2003; Munz et al., 2014), suggesting a potential role for NMDARs localized to RGC terminals. However, pharmacologic studies of retinotopy could not elucidate the neuronal populations in which NMDAR activity was required.

While best studied for their function at the postsynaptic side of the synapse, accumulating evidence suggests that NMDARs may also be expressed presynaptically in many brain regions (Pittaluga and Raiteri, 1990; Aoki et al., 1994; Paoletti et al., 2013; Bouvier et al., 2015, 2018), including visual cortical circuits where they mediate spike-timing-dependent plasticity of connections between neurons in layer (L)4 and L2/3 (Corlew et al., 2007; Bouvier et al., 2018). Previous studies suggest that developing RGCs express NMDARs raising the possibility they could be localized presynaptically in retinorecipient regions (Massey and Miller, 1990; Mittman et al., 1990; Watanabe et al., 1994). Indeed, recent work demonstrated that presynaptic and postsynaptic NMDARs have distinct but complementary roles in developmental plasticity in the retinotectal system (Kesner et al., 2020). However, whether NMDARs expressed by RGCs are required for topographic refinement remains unclear.

To address this, we took a conditional genetic approach to delete the obligate GluN1 subunit of NMDARs in RGCs without altering its expression in retinorecipient regions, allowing us to determine the potential role of presynaptic NMDARs in retinocollicular and retinogeniculate circuit formation. We confirmed a reduction of GluN1 expression in the retina of Chrnb3-Cre;GluN1^flox/flox^ (pre-cKO) mice by *in situ* hybridization and quantitative PCR. Surprisingly, neither the topographic refinement nor the eye-specific segregation of retinal inputs in the SC and dLGN were altered in pre-cKO mice, suggesting a minimal role for NMDAR function in RGCs in these processes. To probe whether NMDARs might be activated presynaptically, we prepared slices of the SC from mice expressing a genetically-encoded GCaMP5 restricted primarily to RGCs. Indeed, administration of NMDA induced modest Ca^2+^ transients, suggesting that NMDARs may be localized presynaptically in the mouse SC. Together, these data suggest a limited role for NMDARs expressed by RGCs in the development and maintenance of ordered projections to image-forming retinorecipient regions.

## Materials and Methods

### Mice

Adult and juvenile mice of either sex were used. Their ages ranged between postnatal day (P)2–P12 or P25–P60. The Chrnb3-Cre transgenic mouse line, described previously (Drayson and Triplett, 2019), was obtained (MMRRC 036469-UCD) and genotyped with two primers against Cre (GTC-CAA-TTT-ACT-GAC-CGT-ACA-CC and GTT-ATT-CGG-ATC-ATC-AGC-TAC-ACC). Mice harboring a floxed allele of the *Grin1* gene (GluN1^flox^) and GCaMP5-IRES-tdTomato reporter mice were generated and genotyped as described previously (Tsien et al., 1996; and Gee et al., 2014). All animals were housed in the research animal facility at Children’s National Research Institute, and all experimental procedures were approved by the Institutional Animal Care and Use Committee.

### Immunohistochemistry

Mice were anesthetized on ice (<P8) or with halothane (2-bromo-2-chloro-1,1,1-trifluoroethane; >P9) and transcardially perfused with ice-cold PBS followed by 4% paraformaldehyde (PFA; pH 7.4). Brains and eyes were dissected and postfixed in 4% PFA at 4°C overnight or 30 min, respectively. After postfixation, brains and eyes were briefly washed in PBS before cryoprotection in 30% (brains) or 10% (eyes) sucrose at 4°C for 24–48 h. Tissues were then embedded in O.C.T. compound (Tissue-Tek 4583) and cooled in a −80°C freezer before being sectioned with ThermoScientific Micron HM 525 cryostat. Sections of 20 μm (brain) and 14 μm (eye) were collected directly onto SuperFrost Gold Plus microscopy slides (Fisher Scientific) and dried overnight at room temperature (RT). Sections were incubated in blocking solution (1% serum, 0.25% Triton X-100) for 1 h at RT and then incubated with primary antibodies diluted in blocking solution at 4°C overnight. The following primary antibodies were used: anti-β3 (Santa Cruz SC-6045, RRID: AB_2065343), anti-Brn-3a (Santa Cruz SC-31984, RRID: AB_2167511), anti-calretinin (Millipore AB1550, RRID: AB_90764), anti-RBPMS (PhosphoSolutions 1830-RBPMS, RRID: AB_2492225), and anti-SatB2 (ABcam AB34735, RRID: AB_2301417). Sections were washed thoroughly in PBS and then incubated with appropriate secondary antibodies (Biotium, RRIDs: AB_2534102, AB_162543) and DAPI diluted in blocking solution for 1 h at RT. Confocal images were acquired at 20× magnification with an Olympus FV1000 microscope, with an Olympus DP71 digital camera attached. Images were analyzed and processed with FIJI (Schindelin et al., 2012). For each genotype, we averaged the number of counted cells over three different retinal sections from each of three animals. Data were analyzed, and graphs were constructed with GraphPad Prism8. All error bars represent the SEM, and statistical analysis was determined using the Mann–Whitney rank-sum test.

### *In situ* hybridization

Tissue was collected and fixed as described above. Complementary DNA for GluN1 [containing nucleotides 25296659–25298429 of the open reading frame (ORF)] was used to make antisense and sense digoxigenin-labeled RNA probes and recognize exons 11–16. Slides were pretreated with PBS at RT for 5 min to rehydrate the slides before being fixed with 4% PFA (pH 7.4) for 15 min. Slides were also pretreated with proteinase K (1 μg/ml) to increase hybridization efficiency. Before being treated with the RNA probe, slides had 300 μl of hybridization buffer (50% formamide, 5× SSC, pH 4.5, 1% SDS, 50 μg/ml yeast tRNA, 50 μg/ml heparin) covered with Parafilm, and incubated at 70°C for 1 h. RNA probes were diluted 1:200 in hybridization buffer and placed onto the slides, covered with Parafilm, and incubated at 70°C overnight. Slides were washed and blocked with TBST/HISS (1× TBS, 1% Tween 20, and 5% HISS) for 1 h at RT. Antibodies against DIG were diluted (1:2000) in TBST/HISS, and 200 μl was placed on each slide for 4°C overnight. Slides were then washed four times for 15 min with TBST and then washed with NTMT three times for 5 min. Slides were then treated with 200 μl of BMPurple (Roche) and developed for 12 h at RT. Images were acquired at 4×, 10×, and 20× magnifications with a brightfield Olympus BX61 microscope.

### Quantitative PCR

Total RNA was isolated from microdissected SC and the whole retina using the Aurum Total RNA Fatty and Fibrous Tissue kit (Bio-Rad #7326830). Synthesis of cDNA was conducted using the iScript Reverse Transcription Supermix for RT-qPCR (Bio-Rad). qPCR was performed on a CFX96 real-time system (Bio-Rad #1708890) in a 20-μl reaction mixture using SsoAdvanced Universal SYBR Green PCR master mix (Bio-Rad). Cycle parameters were 3 s at 95°C and 30 s at 60°C. Data were normalized to housekeeping gene18S. GluN1 primers: 5′-CCAGATGTCCACCAGACTAAA-3′ and 5′-CCATTGACTGTGAACTCCTCTT-3′ (Set 1), 5′-AAGGAGTGGAACGGAATGATG-3′ and 5′-GGCTTGGAGAACTCTATGTACTG-3′ (Set 4), 5′-GTAGCTGGGATCTTCCTCATTT-3′ and 5′-TTCTTCCTCCACACGTTCAC-3′ (Set 5). 18S primers: 5′-CTTTGTCAAGCTCATTTCCTGG-3′ and 5′-TCTTGCTCAGTGTCCTTGC-3′. Data were analyzed using the comparative CT method, and graphs were constructed with GraphPad Prism8 (Schmittgen and Livak, 2008). All error bars represent the SEM, and statistical analysis was determined using an unpaired Student’s *t* test.

### Anterograde RGC axon labeling

Adult mice (P25–P60) were anesthetized by intraperitoneal injection with a ketamine/xylazine cocktail (100/10 mg/kg). Additionally, adult mice were given Buprenex (0.3 mg/kg) for analgesia. Pups (P0–P10) were anesthetized on ice. For focal or bulk labeling of RGCs, a 10% solution of lipophilic dye 1,1’-dioctodecyl-3,3,3’,3’-tetramethylindocarbocyanine perchlorate (DiI) in dimethyleformamide (DMF) or a 2% solution of cholera toxin subunit B (CTB)-488 and CTB-555 in PBS, respectively, was injected using a pulled-glass micropipette attached to a Picospritzer III (Parker-Hannifin). The glass micropipette was inserted into the retina of the anesthetized animal, and ∼100 nl of DiI solution was injected into one eye or ∼500nl CTB-488 was injected into the left eye, and CTB-555 was injected into the right eye. Animals recovered for one week (adults) or 2 d (pups) before being euthanized and their brains postfixed in 4% PFA overnight as described above. The termination zone (TZ) of DiI-labeled RGCs was visualized in whole mount via epifluorescent microscopy. Brains were then embedded in 2–3% agarose and sectioned coronally at 150 μm on a vibratome. The dLGN and SC were visualized at 1.25× magnification via epifluorescent microscopy and analyzed using FIJI.

### Image analysis

To determine topographic refinement of TZs, we calculated a TZ index (TZI), for which the size of the TZ in the SC or dLGN, expressed as a percent of the target area, was divided the injection size, expressed as a percent of the flat-mounted retina. For the dLGN, we quantified the TZI for all sections containing the TZ and determined an average across sections for each animal. The TZIs were statistically analyzed by running a Student’s *t* test with GraphPad Prism8. To assess eye-specific segregation, each coronal section that contained labeled retinal terminals was assessed independently using FIJI, and the average across all sections was used. The boundaries of the dLGN/SC were outlined on a grayscale 8-bit image, and the background was cleared before measuring the size of the dLGN/SC. The areas of ipsilateral and contralateral retinal innervation were determined independently, and the overlapping co-localized pixels were then analyzed using the “AND” function of FIJI’s image calculator. The measurement of the overlap/ipsi area, overlap/total dLGN, and ipsi patch length was then statistically analyzed by running a Student’s *t* test or two-way ANOVA with GraphPad Prism8 for adult and developmental ages, respectively. All error bars represent SEM. Outliers were identified by running a Grubb’s test (outlier test) with GraphPad’s Outlier Calculator and removed from analysis.

### Acute brain slice preparation

Chrnb3-Cre;GCaMP5-tdTomato pups aged P2–P6 of either sex were used. Pups were decapitated, and the brains were rapidly removed and immersed in ice-cold cutting solution (230 mm sucrose, 2.5 mm KCl, 0.5 mm CaCl_2_, 10 mm MgCl_2_, 26 mm NaHCO_3_, 1.25 mm NaH_2_PO_4_, 0.04 mm Na-ascorbate, and 10 mm glucose, pH 7.2–7.4). Coronal and sagittal slices (300 μm) were cut with a vibratome (Leica VT1000S) and transferred to artificial CSF (aCSF; 126 mm NaCl, 4 mm KCl, 2 mm CaCl_2_, 1 mm MgCl_2_, 26 mm NaHCO_3_, 1.25 mm NaH_2_PO_4_, 0.04 mm Na-ascorbate, and 10 mm glucose, pH 7.2–7.4, osmolarity = 310 mOsm/l) bubbled with 95% O_2_ and 5% CO_2_ or into MgCl_2_-free aCSF (126 mm NaCl, 4 mm KCl, 2 mm CaCl_2_, 26 mm NaHCO_3_, 1.25 mm NaH_2_PO_4_, 0.04 mm Na-ascorbate, and 10 mm glucose, pH 7.2–7.4, osmolarity = 310 mOsm/l) bubbled with 95% O_2_ and 5% CO_2_). Slices recovered in oxygenated aCSF for 1 h at RT (21–25°C) before acute slice imaging. During recordings, slices were placed in a perfusion chamber and superfused with oxygenated aCSF at RT for the duration of the experiment. The cells were visualized with a 20× immersion objective (Olympus Optical) and epifluorescence.

### Ca^2+^ imaging and analysis

Ca^2+^ imaging was performed with an Olympus FluoView FVMPE-RS Multiphoton Microscope imaging system using FluoView software and a Ti:Sapphire laser source emitting 140 fs pulses at an 80 MHz repetition rate with a wavelength adjustable for 690–1040 nm (Maitai DeepSee pulsed, infrared laser). Full-field of view images were acquired with XY raster scanning using the 20 × 0.95 NA water-immersion objective. Changes in fluorescence (ΔF) was quantified using ImageJ (NIH) software and expressed as a percentage of baseline (%ΔF/F). Time-lapse images of neuron Ca^2+^ signaling were recorded at a frame rate of 1 Hz. Regions of interest (ROIs) were selected based on the appearance of GCaMP5G Ca^2+^ transients in the time-lapse images. To trigger Ca^2+^ transients, the agonist NMDA (50 or 100 μm) and control K^+^ (5 mm) were dissolved in aCSF and delivered locally by a pressure pulse (10 psi; 100–500 ms) using a Picospritzer III (Parker Instrumentation) while the antagonist MK-801 (10 μm) was delivered via bath perfusion. To avoid potential artifacts because of K^+^ administration, it was always the last agent tested in any given slice. Data were analyzed, and graphs were constructed with GraphPad Prism8. All error bars represent the SEM, and statistical analysis was determined using a one-way ANOVA followed by Tukey’s multiple comparisons test, as indicated in the figure legends; **p < 0.01, ****p < 0.0001, not significant (N.S.) *p* > 0.05.

## Results

### RGC-specific loss of GluN1 expression in Chrnb3-Cre;GluN1^flox/flox^ mice

To study the RGC-specific role of NMDAR function during topographic map formation, we took a conditional genetic approach. We crossed the Chrnb3-Cre line, in which Cre recombinase is expressed broadly by RGCs but not in retinorecipient areas such as the SC or dLGN (Drayson and Triplett, 2019), with a line harboring a floxed allele of the *Grin1* gene coding for the GluN1 subunit of the NMDAR (GluN1^flox^), in which exon 11 through the 3′ end are flanked by loxP sites (Tsien et al., 1996) to generate pre-cKO mice. Previous reports suggest that recombination of this locus results in the complete loss of NMDAR function (Tsien et al., 1996).

**Figure 1.**
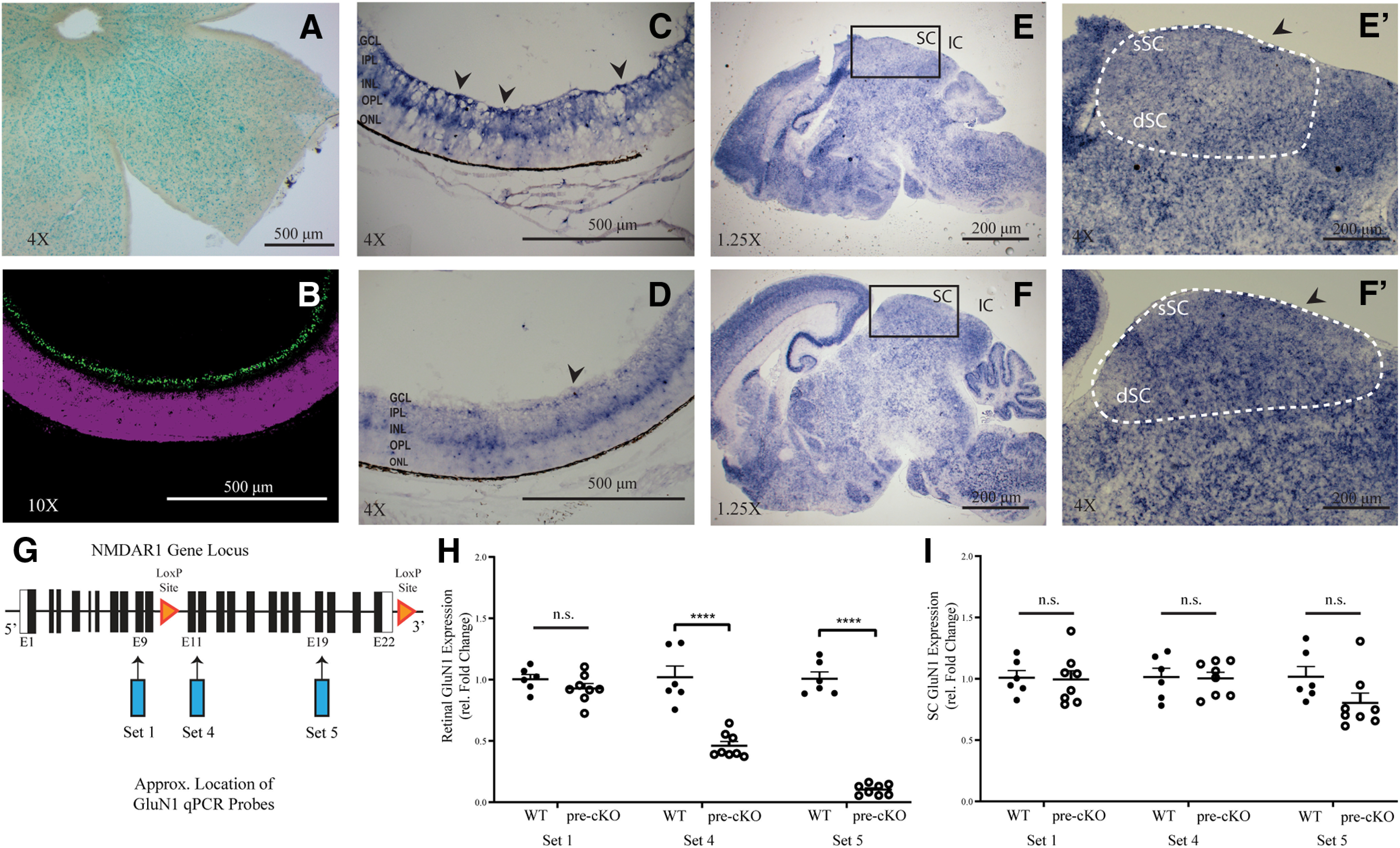
Retina-specific knock-out of GluN1 in Chrnb3-Cre;GluN1^flox/flox^ mice. ***A***, Flat-mounted retina of a Chrnb3-Cre;Rosa^LacZ^ reporter mouse line. ***B***, Section through the retina of a P8 Chrnb3-Cre;Rosa^TdTom^ reporter mouse reveal cells labeled in the GCL (green). ***C***, ***D***, Sections through the retina of P4 Ctl (***C***) and pre-cKO (***D***) mice labeled with GluN1 antisense probe (arrowheads). GCL, ganglion cell layer; IPL, inner plexiform layer; INL, inner nuclear layer; OPL, outer plexiform layer; ONL, outer nuclear layer. ***E***, ***F***, Sagittal sections through the midbrain of P4 Ctl (***E***) and pre-cKO (***F***) mice labeled with GluN1 antisense probe and higher magnification of GluN1 of the superficial and deep layers of the Ctl (***E’***) and pre-cKO (***F’***) SCs. ***G***, Schematic of the GluN1 allele and approximate locations of the different primer sets for qPCR and LoxP sites. ***H***, ***I***, qPCR data for P0 microdissected retina (***H***) and P0 SC (***I***) between Ctl and pre-cKO mice. n.s., not significant; ****p < 0.001.

In the transgenic Chrnb3-Cre mouse line, the majority of expression is observed in the ganglion cell layer (GCL) and distributed broadly across the retina (Drayson and Triplett, 2019). Indeed, we observed that when Chrnb3-Cre mice are crossed with β-galactosidase (LacZ) and tdTomato (tdTom) reporter lines, LacZ expression is observed throughout the retina (Fig. 1*A*), and tdTom expression is restricted to the GCL (Fig. 1*B*). In order to determine whether our genetic strategy was valid, we performed RNA *in situ* hybridization for *GluN1* mRNA in P4 retinal tissue. In control retinas (from mice that were genotyped as Cre^–^ and either GluN1^fl/+^ or GluN1^fl/fl^, [Ctl]), the *GluN1* antisense probe produced a strong signal in cell bodies throughout the GCL and INL (Fig. 1*C*). These findings are consistent with previous reports in which nearly all RGCs expressed the GluN1 subunit (Brandstätter et al., 1994). Strikingly, little to no *GluN1* mRNA expression was detected in the retinas of P4 pre-cKO mice (Fig. 1*D*), suggesting we were able to successfully ablate GluN1 from the GCL in our experimental animals during the first postnatal week. However, some GluN1-expressing cells in the GCL and inner plexiform layer (IPL) were observed (Fig. 1*D*, arrowhead), these cells may be the small population of RGCs not targeted in the Chrnb3-Cre line or displaced amacrine cells (Drayson and Triplett, 2019). Notably, signal from the *GluN1* antisense mRNA probe did label cell bodies throughout the SC of both Ctl and pre-cKO brains (Fig. 1*E*,*F*), suggesting NMDAR function remains intact in this region.

To further demonstrate that *GluN1* expression was reduced in pre-cKO retinas, we performed RT-qPCR comparing the SC and retina of Ctl and pre-cKO animals at P0 using three primer sets (Fig. 1*H*,*I*). Primer Set 1 is located at exon 9, upstream of the flanking site of the GluN1 gene. Primer set four is located near exon 12, just after the flanking site. Primer Set 5 is located near exon 19, toward the end of the GluN1 gene (Fig. 1*G*). This analysis confirmed that our pre-cKO animal had a knock-down of GluN1 expression in the retina with both set 4 and 5 (Set 4 Ctl: 1.020 ± 0.09136, *n* = 6; Set 4 pre-cKO: 0.4604 ± 0.03565, *n* = 8; *p* < 0.001, Student’s *t* test; Set 5 Ctl: 1.007 ± 0.05547, *n* = 6; Set 5 pre-cKO: 0.1029 ± 0.04445, *n* = 8; *p* < 0.001, Student’s *t* test; Fig. 1*H*). As expected, we saw no change in relative expression when analyzing primer Set 1, since the region amplified by this set was not ablated (Set 1 Ctl: 0.8573 ± 0.03889, *n* = 6; Set 1 pre-cKO: 0.9283 ± 0.04024, *n* = 8; *p* = 0.2134, Student’s *t* test; Fig. 1*I*). Additionally, the qPCR analysis with all three primer sets confirmed that GluN1 expression was unchanged in the SC (Set 1 Ctl: 0.8259 ± 0.05876, *n* = 6; Set 1 pre-cKO: 0.7911 ± 0.07140, *n* = 8; *p* = 0.8876, Student’s *t* test; Set 4 Ctl: 0.7815 ± 0.07300, *n* = 6; Set four pre-cKO: 0.8136 ± 0.04889, *n* = 8; *p* = 0.9100, Student’s *t* test; Set 5 Ctl: 0.8128 ± 0.08384, *n* = 6; Set 5 pre-cKO: 0.6149 ± 0.08005, *n* = 8; *p* = 0.0954, Student’s *t* test; Fig. 1*H*). Together, these data indicate neurons in the GCL of the retina express *GluN1* during developmental stages and demonstrate this expression is dramatically reduced in pre-cKO retinas, but not the SC.

### Cytoarchitecture of the retina is unaffected in the absence of GluN1 expression in RGCs

**Figure 2.**
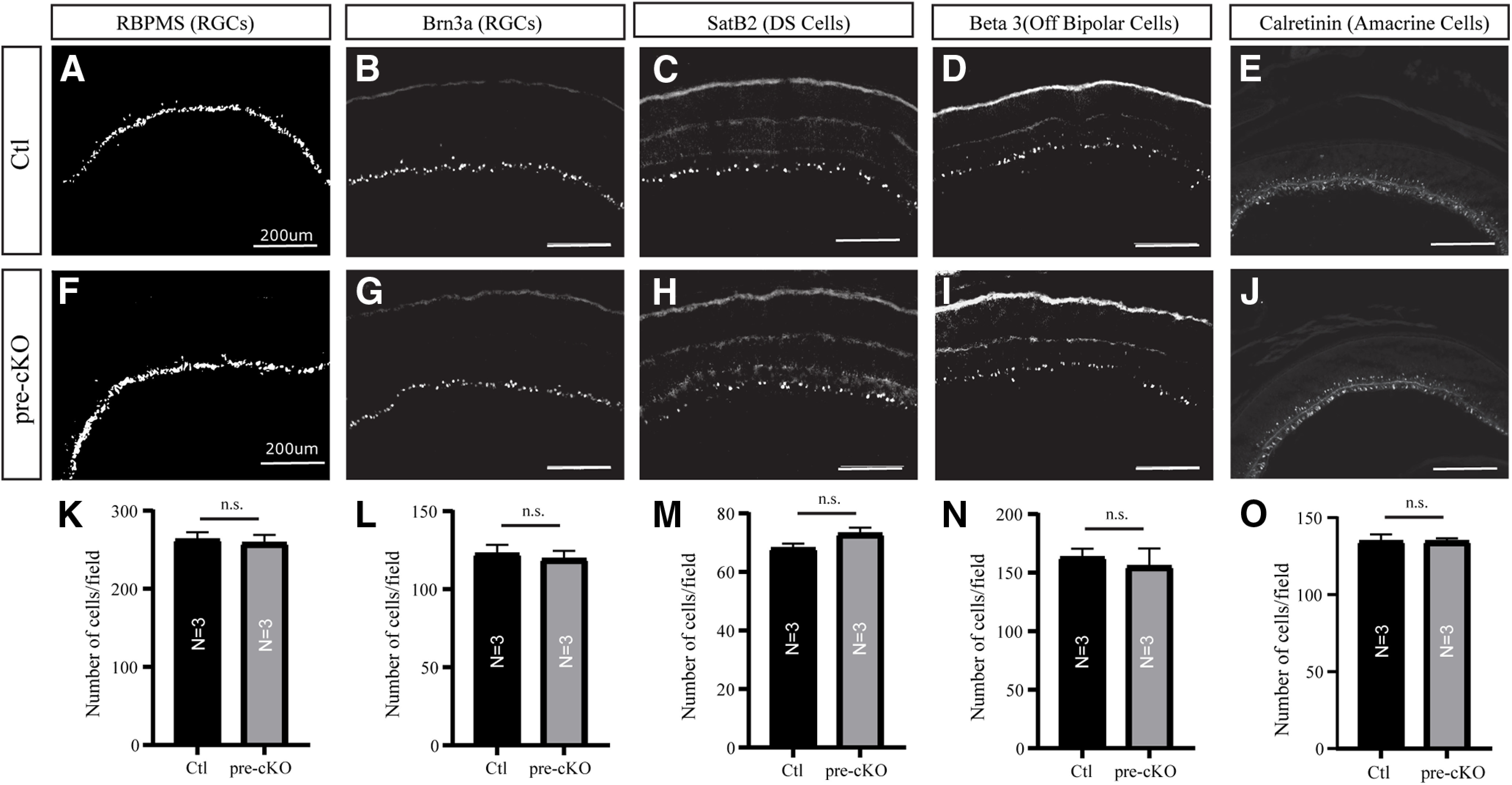
Cytoarchitecture of retina unchanged in Chrbn3-Cre;GluN1^flox/flox^ mice. ***A–J***, Sections through the retinas of P4 Ctl (***A–E***) and pre-cKO (***F–J***) mice stained for RBPMS (***A***, ***F***), Brn3a (***B***, ***G***), SatB2 (***C***, ***H***), β3 (***D***, ***I***), and calretinin (***E***, ***J***). ***K–O***, Quantification of the density of cells labeled with markers used in ***A–J***. n.s., not significant.

Previous studies suggest a critical role for NMDA signaling in the survival of RGCs (Shen et al., 2006). Thus, we wanted to determine whether RGC-specific deletion of GluN1 altered the number of RGCs or morphologic organization of the retina. To begin, we performed immunohistochemistry for markers of different retinal cell types. First, we analyzed the RGC markers RBMPS and Brn3a, since a substantial proportion of Chrnb3-Cre-tagged RGCs expresses Brn3a (Drayson and Triplett, 2019; Fig. 2*A*,*B*,*F*,*G*). The number of RBPMS-labeled cells in a 500 × 500 μm field of the retina for pre-cKO animals (258.7 ± 10.48, *n* = 3) was not significantly different from controls (263.0 ± 9.644, *n* = 3; *p* > 0.9999, Mann–Whitney test; Fig. 2*K*). The number of Brn3a-labeled cells in a 500 × 500 μm field of the retina for pre-cKO animals (122.7 ± 5.239, *n* = 3) was not significantly different from controls (119.3 ± 5.783, *n* = 3; *p* > 0.9999, Mann–Whitney test; Fig. 2*L*). Similarly, we detected no significant difference in the number of cells labeled with SatB2, a putative marker of direction-selective RGCs (Sweeney et al., 2019; Ctl: 68.0 ± 1.732, *n* = 3; pre-cKO: 73.0 ± 2.082, *n* = 3; *p* = 0.2000, Mann–Whitney test; Fig. 2*M*). In order to further examine the morphology of the retina, we stained for β3, a marker for Off bipolar cells, and calretinin, which labels a variety of amacrine cells and RGCs, including their processes in the IPL. As with RBPMS, Brn3a and SatB2, we found no significant difference in number of cells expressing β3 nor calretinin between control and pre-cKO retinas (β3 Ctl: 163.0 ± 7.371, *n* = 3; β3 pre-cKO: 155.3 ± 15.24, *n* = 3; *p* > 0.9999, Mann–Whitney test; calretinin Ctl: 134.7 ± 7.767, *n* = 3; calretinin pre-cKO: 134.7 ± 3.512, n =3; *p* > 0.9999, Mann–Whitney test; Fig. 2*D*,*E*,*I*,*J*,*N*,*O*). Further, the organization of β3 and calretinin-stained processes in the IPL in pre-cKO retinas were grossly organized similarly to those in Ctl retinas. Overall, these data suggest that neither the population of RGCs nor cytoarchitecture of the retina is adversely impacted because of a loss of NMDAR function in RGCs.

### Expression of the GluN1 subunit of the NMDAR in RGCs is not required in topographic refinement

**Figure 3.**
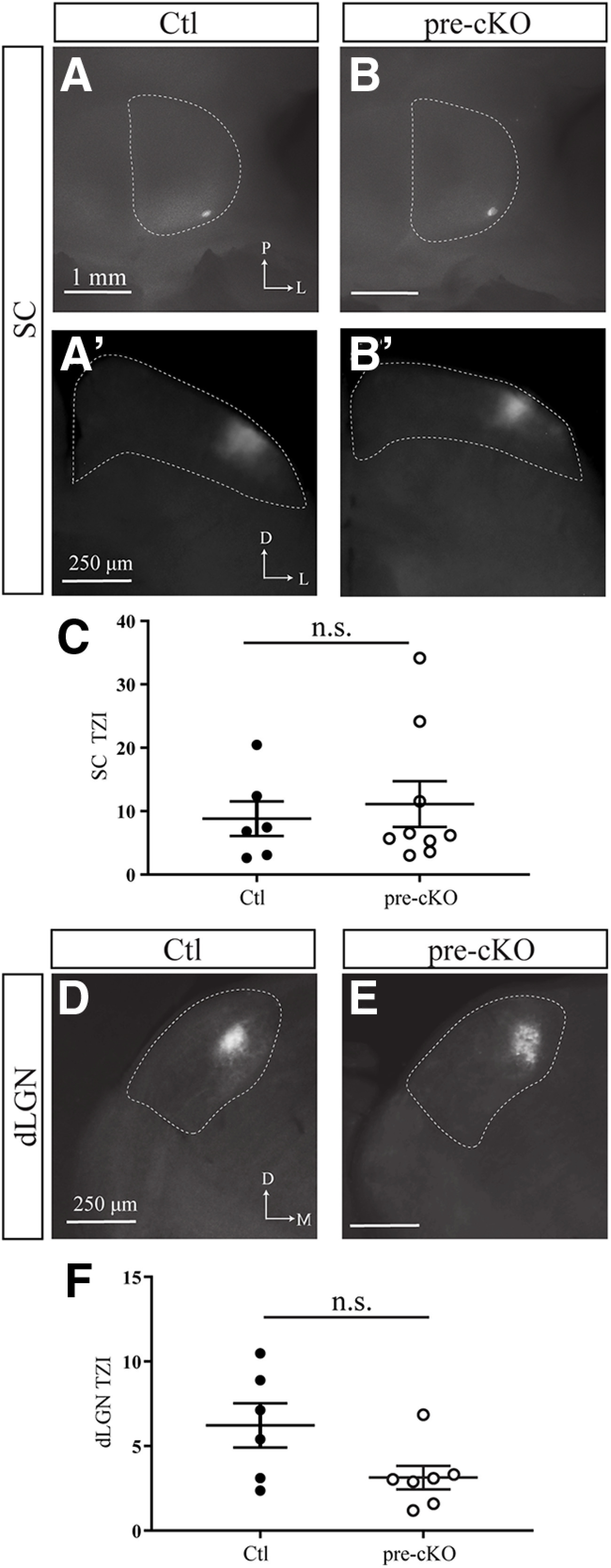
Retinofugal topography is unaltered in Chrnb3-Cre;GluN1^flox/flox^ mice. ***A***, ***B***, Whole-mount fluorescent images of the TZs of labeled RGCs observed in the SC (dashed area) for Ctl (***A***) and pre-cKO (***B***) mice and coronal sections through the corresponding TZs (***A’***, ***B’***). P, posterior; L, lateral; D, dorsal. ***C***, Quantification of the TZIs in the SC. ***D***, ***E***, Coronal sections through the dLGN of Ctl (***D***) and pre-cKO (***E***) mice reveal the TZs of labeled RGCs. ***F***, Quantification of the TZIs in the dLGN. n.s., not significant.

We next tested our hypothesis that NMDAR expression in RGCs is required for the establishment of retinofugal topography using the focal DiI tracing technique in adult animals (P25–P60), as previously described (Kay et al., 2018). In every animal, the axonal projections from the focal DiI injection into the retina showed a topographically appropriate TZ in the SC of adult Ctl and pre-cKO mice (Fig. 3*A*,*B*). Previous studies in which NMDAR function was disrupted pharmacologically suggested that while labeled RGCs terminate in roughly the appropriate topographic zone, the size of the termination field was increased (Simon et al., 1992). To determine whether TZ size was altered in pre-cKO mice, we calculated the TZI by normalizing the TZ size by the injection site size. We observed no change in TZI in mice lacking GluN1 in RGCs (TZI: Ctl: 8.806 ± 2.738, *n* = 6; pre-cKO: 11.13 ± 3.597, *n* = 9; *p* = 0.6481, Student’s *t* test; Fig. 3*C*). Further, we did not observe stray arbors that might indicate subtle deficits not detectable by quantification of TZI (Fig. 3*A’*,*B’*). These data suggest NMDAR function in RGCs is not required for retinocollicular refinement.

In addition to the SC, RGCs project topographically to the dLGN, where spontaneous activity plays a critical role in establishing topography, along with molecular cues (Pfeiffenberger et al., 2006). Therefore, in addition to testing the retinocollicular refinement of our transgenic animals, we observed the retinogeniculate refinement within the same DiI-injected animals. Topographic refinement was not significantly altered, though there appeared to be a trend toward a reduction in TZI in pre-cKO animals (TZI: Ctl: 6.229 ± 1.308; pre-cKO: 3.141 ± 0.6921; *p* = 0.0520, Student’s *t* test; Fig. 3*D–F*). Together, these data suggest expression of GluN1 in RGCs is not required for the development of retinogeniculate map refinement.

### Eye-specific segregation in the SC and dLGN

While retinofugal topography appeared unchanged in the absence of GluN1 expression in RGCs, we reasoned that other developmental processes in visual circuit development that are more reliant on activity-dependent mechanisms may be altered. The segregation of eye-specific inputs in visual areas has served as a classical model to demonstrate the role of both spontaneous activity and visual experience in circuit development and plasticity (Feller, 2009). Indeed, it has been established that during eye-specific segregation in the LGN, large-scale refinement takes place. Glutamatergic waves generated in the retina are critical for maintenance of segregation (Demas et al., 2006), and NMDAR blockade in *in vitro* preparations alters the frequency of glutamatergic waves, but not other attributes, such as velocity (Blankenship et al., 2009). Further, NMDARs have been implicated in the segregation of artificially-induced eye-specific inputs in the frog tectum (Cline and Constantine-Paton, 1990).

**Figure 4.**
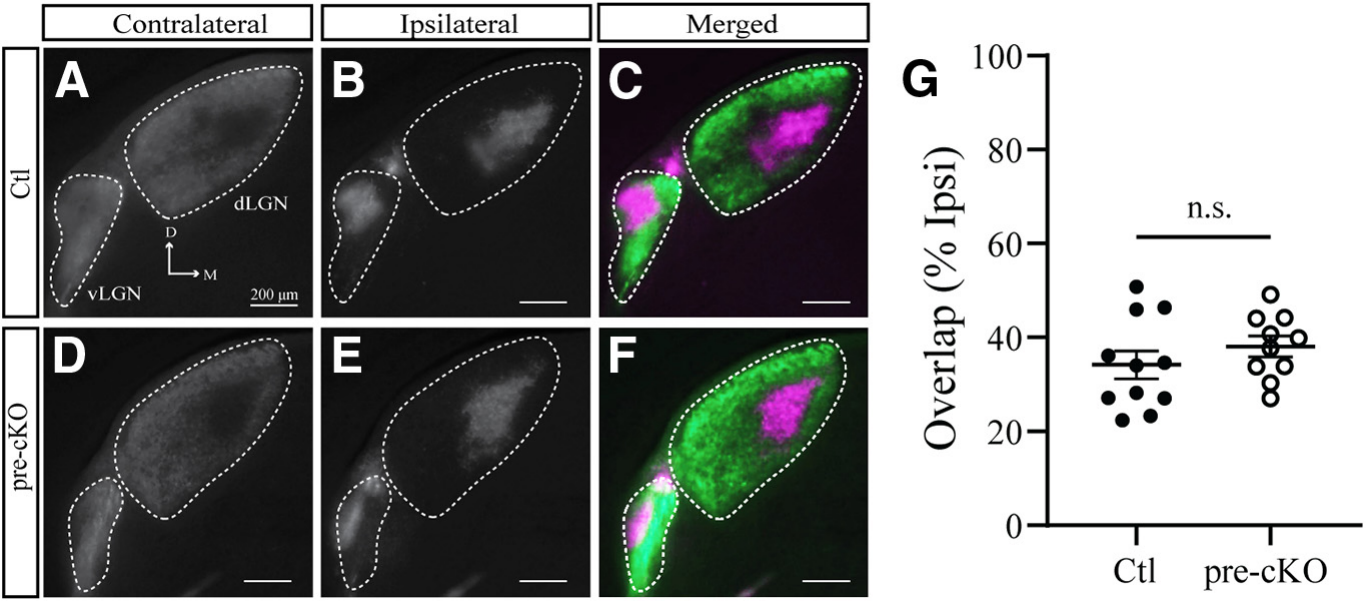
Mature organization of eye-specific segregation in the dLGN is unaltered in Chrnb3-Cre;GluN1^flox/flox^ mice. ***A–F***, Coronal sections through the dLGN of Ctl (***A–C***) and pre-cKO (***D–F***) reveal the terminals of bulk-labeled RGCs originating from the contralateral (***A***, ***D***) or ipsilateral (***B***, ***E***) eye, as well as the degree of overlap (***C***, ***F***). D, dorsal; M, medial. ***G***, Quantification of the amount of overlapping contralateral and ipsilateral inputs to the dLGN. n.s., not significant.

In order to determine whether the expression of GluN1 in RGCs is critical for appropriate eye-specific segregation, we intraocularly injected fluorescently-labeled CTB-488 in the left eye and CTB-555 in the right eye and observed their terminations in the dLGN. As expected, in both control and pre-cKO adults, the majority of the dLGN was occupied by contralateral projections except for the dorsomedial region where the ipsilateral projections terminate (Fig. 4*A–F*). We analyzed eye-specific segregation by calculating the area of overlap between contralateral and ipsilateral projections in relation to the area occupied by ipsilateral projections and found no significant difference between control and pre-cKO adult mice (overlap/ipsi: control: 34.18 ± 2.958, *n* = 11; pre-cKO^:^ 38.08 ± 2.172, *n* = 10; *p* = 0.3090, Student’s *t* test; Fig. 4*G*).

**Figure 5.**
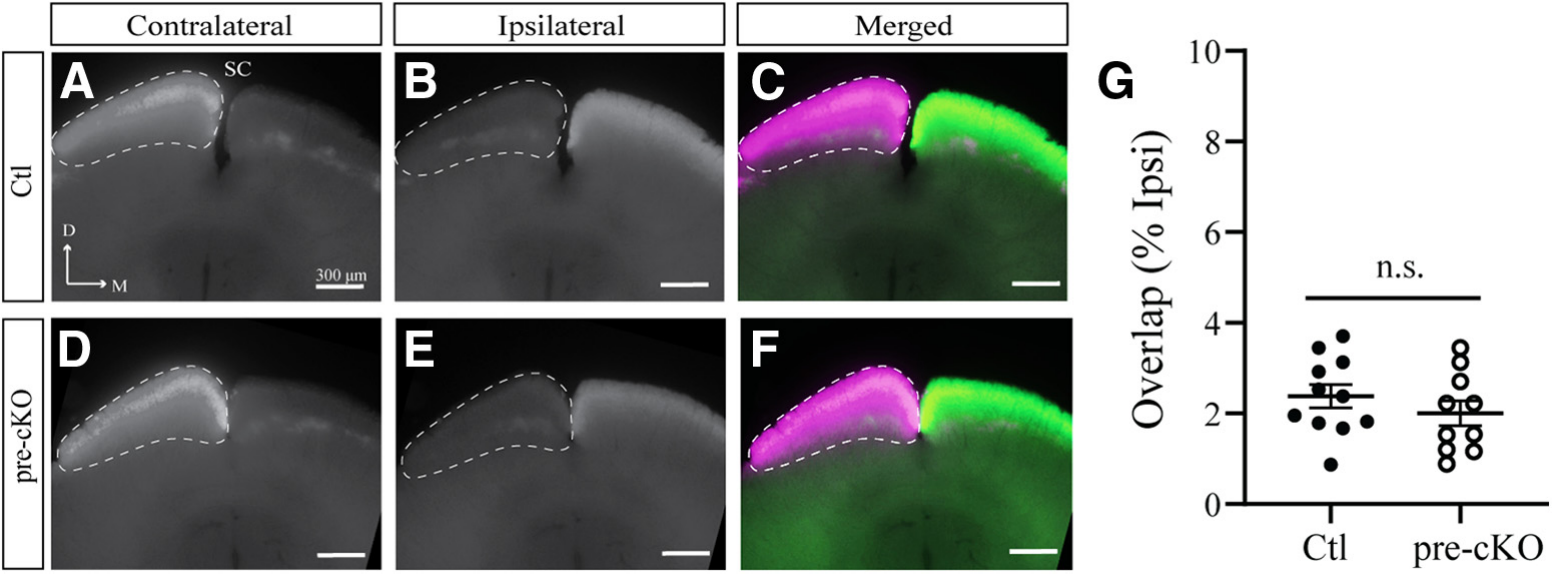
Mature organization of eye-specific segregation in the SC is unaltered in Chrnb3-Cre;GluN1^flox/flox^ mice. ***A–F***, Coronal sections through the SC of Ctl (***A–C***) and pre-cKO (***D–F***) reveal the terminals of bulk-labeled RGCs originating from the contralateral (***A***, ***D***) or ipsilateral (***B***, ***E***) eye, as well as the degree of overlap (***C***, ***F***). D, dorsal; M, medial. ***G***, Quantification of the amount of overlapping contralateral and ipsilateral inputs to the SC. n.s., not significant.

Next, we analyzed the overlap in the SC of these animals and found that eye-specific segregation was not significantly different between the SCs of pre-cKO and control adult animals (overlap/ipsi: Ctl: 2.382 ± 0.2595, *n* = 11; pre-cKO: 2.004 ± 0.2784, *n* = 10; *p* = 0.3320, Student’s *t* test; Fig. 5*A–C*). Although eye-specific segregation is heavily dependent on activity, these data suggest GluN1 expression in RGCs is not required to achieve the mature segregation of eye-specific inputs in the LGN, consistent with previous studies (Hahm et al., 1991; Smetters et al., 1994).

**Figure 6.**
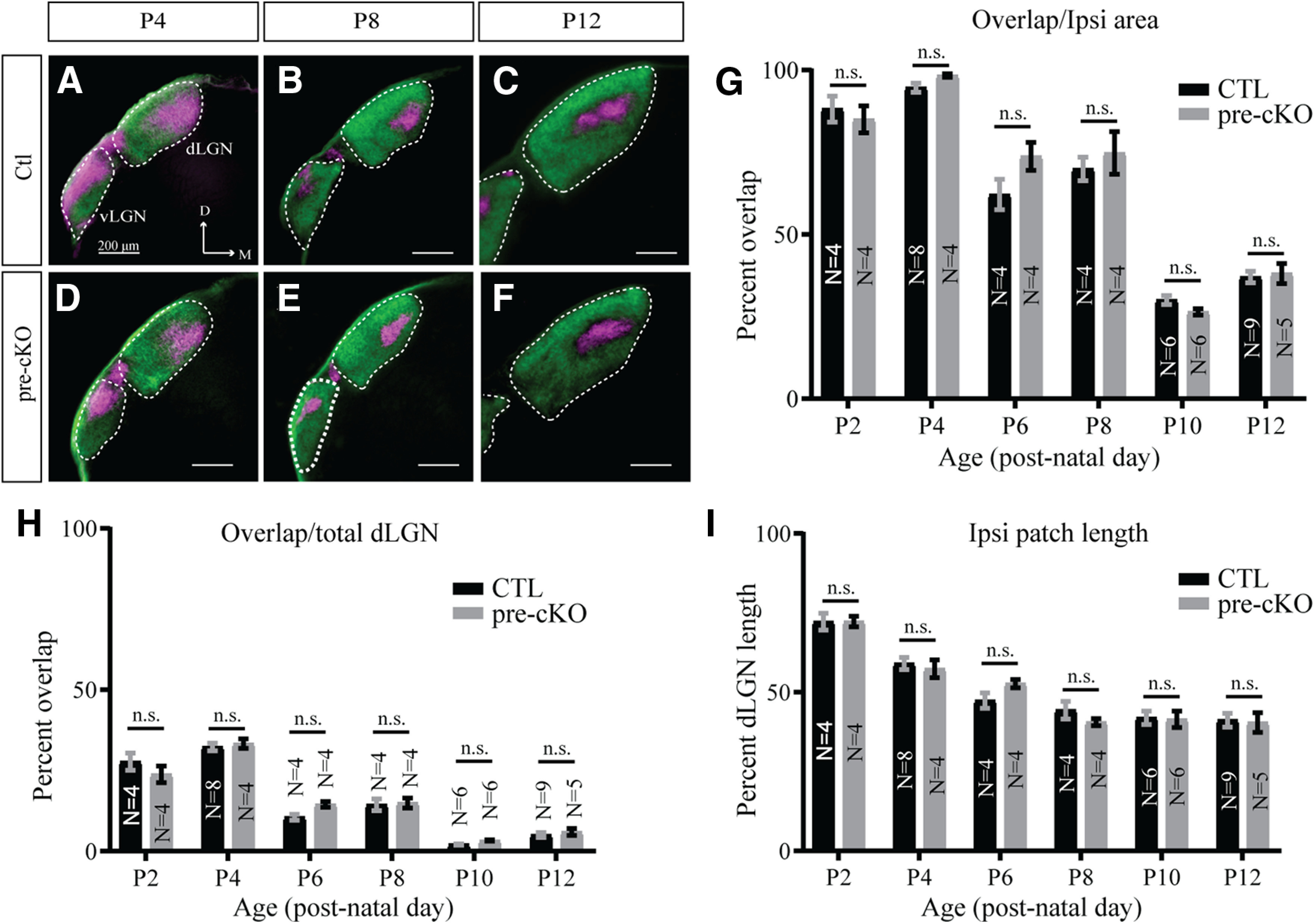
Developmental trajectory of eye-specific segregation in the SC is unaltered in Chrnb3-Cre;GluN1^flox/flox^ mice. ***A–F***, Coronal sections through the SC of Ctl (***A–C***) and pre-cKO (***D–F***) reveal the terminals of bulk-labeled RGCs originating from the contralateral (green) and ipsilateral (magenta) eyes at P4 (***A***, ***D***), P8 (***B***, ***E***), and P12 (***C***, ***F***). D, dorsal; M, medial. ***G***, Quantification of the amount of overlapping contralateral and ipsilateral inputs to the dLGN over the ipsilateral area at the indicated ages in Ctl and pre-cKO mice. ***H***, Quantification of the amount of overlapping contralateral and ipsilateral inputs to the dLGN of Ctl and pre-cKO mice at indicated ages, presented as the percent of the total dLGN area. ***I***, Quantitative comparison of the ipsilateral patch length between Ctl and pre-cKO at indicated ages, expressed as the percentage of the dLGN length covered by the ipsilateral patch along the DM-VL axis. n.s., not significant.

We next wondered whether the developmental trajectory of eye-specific segregation might be altered in the absence of GluN1 expression in RGCs. We tested this possibility by analyzing the overlap of contralateral and ipsilateral projections in pups (P2–P12; Fig. 6). At P4, the retinogeniculate axons from the two eyes are not well separated, as expected (Fig. 6*A*,*D*). However, over time, eye-specific segregation became more and more refined by the end of the second postnatal week (Fig. 6*B*,*C*,*E*,*F*). As expected, we found a main effect of age in the amount of overlap between ipsi-RGC and contra-RGC terminals in the dLGN, when calculated as a proportion of the ipsi-RGC domain (*p* < 0.0001, two-way ANOVA). Interestingly, we found that overlap was different between each age group (*p* < 0.05, Tukey’s multiple comparisons test) except for between P6 and P8 (*p* = 0.7511). These data suggest paradoxical increases in overlap from P2 to P4 and P10 to P12 in our dataset, but the general trend is a decrease in overlap over time, consistent with previous data (Pfeiffenberger et al., 2005). However, we did not find any effect of genotype (*p* = 0.1657, two-way ANOVA) nor any interaction between age and genotype (*p* = 0.1516, two-way ANOVA; Fig. 6*G*). We next wondered whether the increase in overlap was because of changes in the size of the ipsi-RGC domain in the dLGN. To do so, we calculated the amount of overlap of ipsi-RGC and contra-RGC terminals as a proportion of the total size of the dLGN. Again, we observed a main effect of age (*p* < 0.0001, two-way ANOVA), but found no effect of genotype (*p* = 0.4183) nor any interaction between age and genotype (*p* = 0.1981; Fig. 6*H*). Interestingly, we found significant differences between all age groups (*p* < 0.0001, Tukey’s multiple comparisons test) except when comparing P6 to P8 (*p* = 0.6827) and P10 to P12 (*p* = 0.1773). Lastly, we analyzed the ipsilateral patch length over the length of the dLGN (Fig. 6*I*). Similar to our analyses of overlap, we found a main effect of age (*p* < 0.0001, two-way ANOVA), but not genotype (*p* = 0.8871) nor any interaction the two (*p* = 0.6740). For this metric, we found significant differences when comparing P2 or P4 to all ages (*p* < 0.05), when comparing P6 to P10 and P12 (*p* < 0.05), and a trend toward a difference when comparing P6 to P8 (*p* = 0.0740). Altogether, these data suggest NMDAR activity in RGCs is not required for the development or maintenance of eye-specific segregation, but reveal age-dependent effects on eye-specific segregation.

### Activation of NMDA-mediated response in RGC terminals in retinorecipient areas

**Figure 7.**
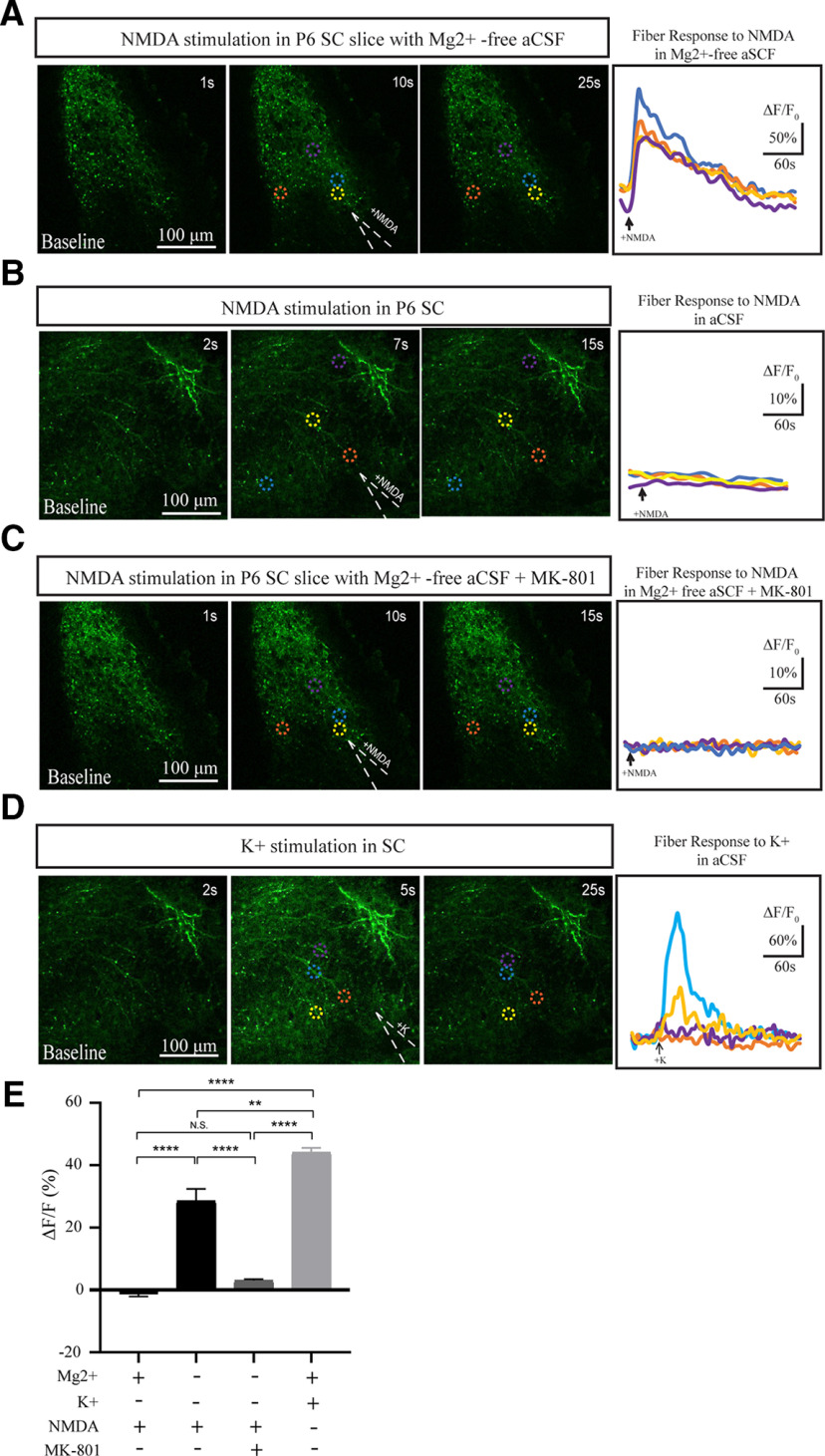
NMDA elicited Ca^2+^ transients in the terminals of developing RGCs. ***A–D***, Time series fluorescent imaging of Ca^2+^ response in RGC terminals elicited in acute brain slices from P6 Chrnb3-Cre;GCaMP5::TdTom mice by focal application of NMDA in the SC in Mg^2+^-free aCSF (***A***), NMDA stimulation in the SC in aCSF (***B***), NMDA stimulation in the SC in Mg^2+^-free aCSF + MK-801 (***C***), and K^+^ stimulation in the SC in aCSF (***D***) from a glass pipette (white dashes in center panel). Graphs of change in fluorescence over time at four different regions of interest (dashed colored circles) reveal the response elicited under each condition (right panels). ***E***, Quantification of the peak ΔF/F (%) in the indicated conditions. **p < 0.01; ****p < 0.0001; n.s., not significant.

Thus far, our data suggest that retinofugal development is not dependent on NMDAR expression in RGCs. One possibility for this could be that presynaptic NMDARs may not be located in the terminals of RGCs in these retinorecipient centers, contrary to what has been suggested in the frog tectum (Kesner et al., 2020). To test this, we crossed the Chrnb3-Cre line with Cre-dependent GCaMP5G::tdTom reporter mice (Gee et al., 2014) and performed Ca^2+^ imaging in retinorecipient areas, where only RGC terminals would be labeled. We began by testing the activity of RGC axons in the SC with NMDA at 50 mm and 100 mm, and found that an elicited calcium response could be visualized in Mg^2+^-free aCSF, but not in aCSF (NMDA in Mg^2+^-free aCSF: 28.48 ± 3.962, *n* = 6; NMDA in aCSF: −3.907 ± 0.9898, *n* = 5, *p* < 0.0001, Tukey’s *post hoc* test; Fig. 7*A–C*,*E*). Importantly, this response was significantly smaller than that observed when we administered 5 mm K^+^ stimulation as a positive control (K^+^ stimulation in aCSF: 44.10 ± 1.482, *n* = 3, *p* = 0.0060, Tukey’s *post hoc* test; Fig. 7*D*,*E*), suggesting that NMDA application was not driving wholesale activation of RGCs, but rather specific activation of NMDARs on RGC terminals. Indeed, when we administered 50 mm and 100 mm of NMDA in the presence of the specific NMDAR antagonist, MK-801, the calcium response was ablated (NMDA in Mg^2+^-free aCSF + MK-801: 3.057 ± 0.3887, *n* = 6, *p* < 0.0001, Tukey’s *post hoc* test; Fig. 7*C*,*E*). We observed a similar pattern of activation of RGC terminals in the dLGN (data not shown), consistent with previous data demonstrating that most RGCs that project to the dLGN also project to the SC (Dhande et al., 2011). Overall, these data suggest that NMDA-mediated activity is elicited by direct stimulation of RGC terminals in the SC, supporting the presence of presynaptic NMDARs at retinocollicular synapses.

## Discussion

During the development of the visual system, RGCs undergo extensive remodeling mediated by a combination of molecular cues, axon-axon competition and neuronal activity to develop precise terminations in retinorecipient regions. Spontaneous activity in the form of retinal waves helps ensure retinocollicular and retinogeniculate refinement; however, the specific mechanisms by which activity mediates these processes remain unclear. Here, we tested the role of NMDARs expressed by RGCs in the development of precise retinofugal projection organization in the mouse. We found that our novel conditional genetic approach successfully ablated NMDARs from RGCs without altering gross retinal organization or expression in the SC. Anatomical tracing experiments revealed no changes in topographic refinement nor eye-specific segregation in either the SC or dLGN. This is the case despite the fact that we observed NMDA-elicited Ca^2+^ transients in RGC terminals in retinorecipient regions. Together, these data demonstrate that NMDARs expressed by RGCs are not required for topographic refinement nor eye-specific segregation in image-forming retinorecipient regions

### NMDARs expressed by RGCs are not required for retinotopy

Previous studies demonstrated that neuronal activity mediated through NMDARs plays an important role in establishing the topography of retinocollicular projections. When NMDARs were chronically blocked during SC development, mRNA levels of GluN1 are decreased (Hofer et al., 1994); additionally, proper activity level of NMDARs is required for the appropriate development and refinement of the retinofugal map (Cline and Constantine-Paton, 1989; Simon et al., 1992; King et al., 1996; Mize and Butler, 2000). Further, terminal arbors of RGCs in the optic tectum exhibit robust dynamics, which are disrupted when NMDAR function is blocked (Rajan et al., 1999; Ruthazer et al., 2003; Munz et al., 2014). And NMDARs play a critical role in the plasticity required for synaptic convergence following map compression in the SC (Huang and Pallas, 2001). However, these pharmacological studies could not distinguish between potential contributions of presynaptic or postsynaptic NMDAR activity in these processes, each of which have been implicated in plasticity in other brain regions (Paoletti et al., 2013).

To overcome this limitation, we developed a conditional knock-out model to directly determine whether NMDARs expressed by RGCs play a role in map formation. In the SC of pre-cKO mice, we did not observe alterations in the size of TZs from labeled RGCs, demonstrating that NMDARs expressed by these neurons are not required for topographic refinement in this region. Interestingly, these data are somewhat inconsistent with recent work in which sparser retinotectal terminals were observed when GluN1 expression was knocked down specifically in RGCs (Kesner et al., 2020). However, the total size of the terminal arbor may have been less dramatically impacted, as no change was observed in total terminal branch length. This result is consistent with previous studies leveraging pharmacologic NMDAR blockade, in which disruptions of RGC axonal arbor dynamics were observed (Rajan et al., 1999; Ruthazer et al., 2003; Munz et al., 2014), but the overall mature organization of arborizations was not dramatically altered (Cline and Constantine-Paton, 1989). Indeed, functional analyses revealed no changes in overall receptive field size when GluN1 expression was knocked down in RGCs, consistent with minimal change in topography (Kesner et al., 2020). One possible reason for a lack of phenotype in terms of topographic refinement may be that the Cre line chosen is expressed in only ∼65% of RGCs (Drayson and Triplett, 2019). However, our qPCR data suggest a substantial knock-down of all retinal expression of *GluN1*, suggesting that the vast majority of RGCs lack expression in this model. Intriguingly, we did observe a trend toward a decreased TZI for retinogeniculate projections, which would be consistent with sparser terminals reported for retinotectal projections lacking NMDARs in RGCs (Kesner et al., 2020). This raises the intriguing possibility that presynaptic NMDARs may play distinct roles in circuit formation in the SC and dLGN. However, more sophisticated analyses of retinal convergence in the SC and dLGN are needed to draw firm conclusions regarding the context-dependent roles of presynaptic NMDARs.

### NMDARs in RGCs are not required for eye-specific segregation

The establishment of eye-specific organization in the visual system has served as a model to understand the mechanisms underlying circuit development and plasticity (Arroyo and Feller, 2016; Hensch and Quinlan, 2018). Intriguingly, previous studies in which eye-specific segregation was induced in the context of retinotectal projections in frogs (Constantine-Paton and Law, 1978) suggested activation or inhibition of NMDAR function could enhance or disrupt segregation, respectively (Cline and Constantine-Paton, 1990). Here, we found no changes in the mature organization of eye-specific lamina in the SC nor dLGN when NMDAR function was disrupted in RGCs. Although, as noted for the lack of phenotype observed for retinocollicular projections, the fact that not all RGCs are targeted in Chrnb3-Cre mice could mask a potential role for presynaptic NMDARs in eye-specific segregation. Further, we did not observe alterations in the developmental trajectory of segregation in the dLGN between genotypes in any of our analyzed parameters. Interestingly, we did observe increases in overlap as a percent of the ipsi-RGC domain from P2 to P4 and P10 to P12. The increased overlap from P2 to P4 may be driven by the fact that ipsi-RGC innervation of the dLGN is not complete until P4 (Godement et al., 1984). One explanation for the increase overlap observed between P10 to P12 could be a reduction in the number ipsi-RGC before eye opening. Indeed, when we analyzed overlap as a proportion of the size of the dLGN, we did not observe significant differences between P10 and P12. However, we found that the size of the ipsi-RGC patch, as measured by its length, decreased until P8, but not thereafter. Together, these data support a slight reversal of eye-specific segregation just before eye-opening, which could be masked depending on the method of quantification. Of note, many investigations of eye-specific segregation in the mouse dLGN did not sample with the frequency that we did (every 2 d) (Muir-Robinson et al., 2002; Jaubert-Miazza et al., 2005; Demas et al., 2006). One study that sampled with the same frequency did not report overlap at P2 or P12 (Pfeiffenberger et al., 2005). Thus, the changes we observe may reflect a high degree of dynamics in eye-specific sorting in the mouse dLGN. Though it is important to note that the changes we observed were small, and the general trend was consistent with these previous studies.

In addition to roles in synaptic plasticity, axonal refinement, and arbor stabilization, NMDARs have also been implicated in the generation of glutamatergic waves, which play a critical role in the maintenance of eye-specific segregation (Cline and Constantine-Paton, 1989; Iwasato et al., 1997; Ruthazer and Cline, 2004; Hu et al., 2005; Demas et al., 2006; Munz et al., 2014). Indeed, NMDAR blockade in *in vitro* preparations alters the frequency of glutamatergic waves, but not other attributes, such as velocity (Blankenship et al., 2009). These results raise the possibility that disruption of NMDAR expression in RGCs might alter the pattern of glutamatergic waves. While we did not monitor these waves directly, the lack of an eye-specific segregation phenotype observed in pre-cKO mice, both in the mature and developing state, suggests NMDARs in RGCs are dispensable for the wave-dependent information mediating maintenance of segregation. These findings are consistent with recent work elucidating a role for NMDARs on the presynaptic side of bipolar cell terminals in the initiation and propagation of glutamatergic waves (Zhang et al., 2016b).

### NMDARs may be present on developing RGC terminals

The lack of disruptions in topography and eye-specific segregation we observed in pre-cKO mice raised the question of whether, in fact, NMDARs are localized and functional in terminals of developing RGCs. The expression pattern of NMDARs in the developing brain has been difficult to examine because of a lack of suitable antibodies for immunolocalization of the obligate GluN1 subunit. While, recent studies suggest NMDARs are expressed in both the cell bodies and dendrites of RGCs (Zhang et al., 2016a), the successful labeling of NMDARs on axons or at terminals has not been reported in murine models. To explore the possibility of NMDARs located presynaptically in murine RGC terminals, we used combination Chrnb3-Cre;GCaMP5::TdTom mice. This animal model allows us to not only visualize RGC terminals in slices through retinorecipient regions, but it also restricts Ca^2+^ indicator expression to RGC terminals. Using this methodology, we observed Ca^2+^ transients in RGC terminals in both the dLGN and the SC, which were ablated in the presence of the NMDAR-specific antagonist, MK-801. These data suggest that NMDARs are localized presynaptically. However, the possibility that indirect activation of RGC terminals occurs via administration of NMDA cannot be ruled out, as the temporal dynamics of GCaMP are too slow to resolve this. However, the presence of axo-axonal synapses onto RGCs that could lead to such a result have not been reported to our knowledge.

In conclusion, we have used a conditional genetic knock-out method to probe the role of NMDARs expressed by RGCs in the development of ordered connectivity in image-forming retino-recipient nuclei. We did not observe alterations in either topography or eye-specific segregation in the SC or dLGN in pre-cKO mice, demonstrating that NMDARs in RGCs play a minimal role in these processes. Further, using conditional expression of genetically-encoded Ca^2+^ indicators in RGCs, we present evidence that NMDARs may be present in developing RGC terminals in the mouse.

## Synthesis

Reviewing Editor: Lynda Erskine, University of Aberdeen Decisions are customarily a result of the Reviewing Editor and the peer reviewers coming together and discussing their recommendations until a consensus is reached. When revisions are invited, a fact-based synthesis statement explaining their decision and outlining what is needed to prepare a revision will be listed below. The following reviewer(s) agreed to reveal their identity: Edward Ruthazer.

Your manuscript has now been re-reviewed and I am pleased to inform you that, barring some minor text and figure changes, it is now suitable for publication in eNeuro. Both reviewers are agreed that the revised manuscript has been improved substantially and that this is a nicely executed study that addresses the question of the importance of RGC NMDARs in retinotopic map formation and eye specific input segregation. While the main findings are negative results the tools used are nicely validated. The main concern is that one of the “negative” findings is in fact extremely close to being statistically significant and may instead be a bit underpowered due to some variability in the experimental population. This needs to be recognized in the text and conclusions of the paper. There are also some additional points raised in the reviews all of which should be able to be addressed with small changes to the text or figures.

Below is the list of points that we are all agreed should be addressed prior to publication:

1. Line 307 “Again, we found that topography appeared unchanged, and the size of TZs was unaffected” is too strong a statement when you have a fairly large effect size with p = 0.0520. Instead, you could say “Topographic refinement was not significantly altered, though there appeared to be a trend toward a reduction in TZI in pre-cKO animals”.

2. Line 411 you claim your data are somewhat inconsistent with the findings of Kesner et al., and this appears correct in the colliculus. However the near-significant trend toward smaller TZs in the pre-cKO dLGN seems rather compatible. Rather than making too big a deal of the “lack of significance” here it would be valuable to convey the possibility that your findings of a trend toward a smaller TZ may constitute a partial validation of the findings of Kesner et al in the tadpole, but also highlights subtle species-specific differences.

3. Fig.4G and 5G are the same so there must have been a mistake when redoing the figures.

4. The authors have added a sentence line 421-422 “One possible reason for a lack of phenotype in terms of topographic refinement may be that the Cre line chosen is expressed in only ∼80% of RGCs (Drayson & Triplett, 2019)”. This is probably a little overestimated as Brn3a+ cells represent ∼80% of RGCs and not all reporter cells are Brn3a+.

5. The argument about the Cre line used and the number of RGCs affected that could be insufficient to affect topographic refinement is also valid for the eye-specific segregation and should be mentioned in the corresponding paragraph or be a global comment that ponder all these results.

6. The observation of the increased overlap from P2 to P4 and from P10 to P12 in Fig.6 is not that surprising. At P2, ipsilateral fibers are still invading the dLGN and this process is completed by P3/P4 and the eye-specific segregation process starts from P4 (Godement et al.,1984), so this could explain why the authors have an increased overlap from P2 to P4 as the ipsilateral axons are still growing in the dLGN and the segregation process has not really started yet. Concerning the increased overlap from P10 to P12, as this overlap is expressed as a percentage (number of overlapped pixels/ total number of ipsilateral pixels if I am correct), this increase in percentage could be also linked to a reduction in the number of ipsilateral pixels rather than an increased overlap. This data is sometimes also expressed as a number of overlapped pixels/total number of contralateral pixels or dLGN size (Jaubert-Miazza et al.,2005; Pfeiffenberger et al., 2005). It would be interesting to compare the raw values of the number of ipsilateral pixels, contralateral pixels and overlapped pixels and use also these different parameters for percentage as was done in Figure 4 of Pfeiffenberger et al., 2005. There is no such quantitative data to chronicle the detailed developmental steps of ipsilateral and contralateral innervation in the literature yet and it would be interesting to have it as a reference.

7. Fig.6A and D says P4 while the text state P2 when referring to the same images.

8. The contrast of some panels in Figures 4 and 5 could be improved.

9. Around line 182 you should indicate that outliers were identified using X test and removed from analysis.

10. Minor text errors:

Line 13: no SIGNIFICANT changes

Line 14: suggesting NMDAR function in RGCs is not an absolute requirement for topographic refinement.

Line 18: retinofugal TOPOGRAPHIC map formation

Line 22: “is critical” “is thought to be critical. (I don't think it has been properly shown).

Line 31 is the vLGN and IGL not topographically organized? I am unsure you can safely say TWO image forming regions.

Line 284: ...suggest that NEITHER the population of RGCs nor cytoarchitecture of the retina IS ADVERSELY impacted...”

Line 319 critical FOR

Line 343 overlap “OF CONTRALATERAL and ipsilateral projections IN pups”?

Line 348 presumably the Tukey's test followed a one-way ANOVA (otherwise it would not be permitted with no significant interaction in the two-way ANOVA). This should be clarified.

## Author Response

Point-by-point response to Reviewers:

We thank the reviewers for their reading and critiques of the resubmitted manuscript.

Below we address specific points and our response to each critique in italicized font.

Below is the list of points that we are all agreed should be addressed prior to

publication:

1. Line 307 “Again, we found that topography appeared unchanged, and the size of TZs

was unaffected” is too strong a statement when you have a fairly large effect size with p

= 0.0520. Instead, you could say “Topographic refinement was not significantly altered,

though there appeared to be a trend toward a reduction in TZI in pre-cKO animals”.

We agree that the statement is too strong with a p value of 0.0520. We have

modified the text to instead read “Topographic refinement was not significantly altered,

though there appeared to be a trend toward a reduction in TZI in pre-cKO animals” as

suggested. (lines 309-10).

2. Line 411 you claim your data are somewhat inconsistent with the findings of Kesner

et al., and this appears correct in the colliculus. However the near-significant trend

toward smaller TZs in the pre-cKO dLGN seems rather compatible. Rather than making

too big a deal of the “lack of significance” here it would be valuable to convey the

possibility that your findings of a trend toward a smaller TZ may constitute a partial

validation of the findings of Kesner et al in the tadpole, but also highlights subtle

species-specific differences.

We appreciate this point and added the following text to highlight potential

differences in the role of pre-synaptic NMDARs in the SC vs. dLGN: “Intriguingly, we did

observe a trend towards a decreased TZI for retinogeniculate projections, which would

be consistent with sparser terminals reported for retinotectal projections lacking

NMDARs in RGCs (Kesner et al., 2020). This raises the intriguing possibility that presynaptic NMDARs may play distinct roles in circuit formation in the SC and dLGN.

However, more sophisticated analyses of retinal convergence in the SC and dLGN are

needed to draw firm conclusions regarding the context-dependent roles of pre-synaptic

NMDARs.” (lines 441-7).

3. Fig.4G and 5G are the same so there must have been a mistake when redoing the

figures.

We appreciate the reviewers' attention to detail and apologize for the mistake.

We have updated Fig. 5G with the correct data.

4. The authors have added a sentence line 421-422 “One possible reason for a lack of

phenotype in terms of topographic refinement may be that the Cre line chosen is

expressed in only ∼80% of RGCs (Drayson & Triplett, 2019)”. This is probably a little 2

overestimated as Brn3a+ cells represent ∼80% of RGCs and not all reporter cells are

Brn3a+.

We have updated the text to reflect a more accurate estimate of the proportion of

RGCs that are labeled (∼65% or ∼80% of 80%). (line 439).

5. The argument about the Cre line used and the number of RGCs affected that could

be insufficient to affect topographic refinement is also valid for the eye-specific

segregation and should be mentioned in the corresponding paragraph or be a global

comment that ponder all these results.

Good point. We have added the following sentence to the discussion of eye-specific segregation: “Although, as noted for the lack of phenotype observed for

retinocollicular projections, the fact that not all RGCs are targeted in Chrnb3-Cre mice

could mask a potential role for pre-synaptic NMDARs in eye-specific segregation.” (lines

457-59).

6. The observation of the increased overlap from P2 to P4 and from P10 to P12 in Fig.6

is not that surprising. At P2, ipsilateral fibers are still invading the dLGN and this

process is completed by P3/P4 and the eye-specific segregation process starts from P4

(Godement et al.,1984), so this could explain why the authors have an increased

overlap from P2 to P4 as the ipsilateral axons are still growing in the dLGN and the

segregation process has not really started yet. Concerning the increased overlap from

P10 to P12, as this overlap is expressed as a percentage (number of overlapped pixels/

total number of ipsilateral pixels if I am correct), this increase in percentage could be

also linked to a reduction in the number of ipsilateral pixels rather than an increased

overlap. This data is sometimes also expressed as a number of overlapped pixels/total

number of contralateral pixels or dLGN size (Jaubert-Miazza et al.,2005; Pfeiffenberger

et al., 2005). It would be interesting to compare the raw values of the number of

ipsilateral pixels, contralateral pixels and overlapped pixels and use also these different

parameters for percentage as was done in Figure 4 of Pfeiffenberger et al., 2005. There

is no such quantitative data to chronicle the detailed developmental steps of ipsilateral

and contralateral innervation in the literature yet and it would be interesting to have it as

a reference.

We thank the reviewers for their insight and have included the suggested

analyses of overlap as a percent of whole dLGN. Interestingly, we find that the increase

in overlap remains significant from P2 to P4, but not from P10 to P12, when calculated

in this manner. However, this was not driven by a decrease in ipsi-RGC inputs, as ipsi

patch length did not change between P10 and P12. These data are presented in Fig. 6H

& I and discussed in the results section (lines 357-72). In addition, we added text to the

discussion suggesting that the increase from P2 to P4 may be due to continued

innervation of the dLGN by ipsi-RGCs, and suggest that a slight reversal of eye-specific

segregation may occur prior to eye opening.” (lines 462-71).

7. Fig.6A and D says P4 while the text state P2 when referring to the same images.3

We thank the reviewer for pointing out this typo and have fixed it to say P4 in the

text. (line 346).

8. The contrast of some panels in Figures 4 and 5 could be improved.

We have improved contrast on these figures.

9. Around line 182 you should indicate that outliers were identified using X test and

removed from analysis.

We agree and have added the following sentence to the text: “Outliers were

identified by running a Grubb's test (outlier test) with GraphPad's Outlier Calculator and

removed from analysis.” (lines 183-84).

10. Minor text errors:

Line 13: no SIGNIFICANT changes

We agree and have added “significant” to the text.

Line 14: suggesting NMDAR function in RGCs is not an absolute requirement for

topographic refinement.

We have updated the text to read “suggesting NMDAR function in RGCs is not

an absolute requirement for topographic refinement.”

Line 18: retinofugal TOPOGRAPHIC map formation

We have updated the text to include “topographic.”

Line 22: “is critical” “is thought to be critical. (I don't think it has been properly shown).

We have updated the text to include “thought to be” critical.

Line 31 is the vLGN and IGL not topographically organized? I am unsure you can safely

say TWO image forming regions.

We have updated the text to say “two main image-forming regions.”

Line 284: ...suggest that NEITHER the population of RGCs nor cytoarchitecture of the

retina IS ADVERSELY impacted...”

We have updated the text to read “Overall, these data suggest that neither the

population of RGCs nor cytoarchitecture of the retina is adversely impacted due to a

loss of NMDAR function in RGCs.”4

Line 319 critical FOR

We have fixed this typo.

Line 343 overlap “OF CONTRALATERAL and ipsilateral projections IN pups”?

We have updated the text to read “We tested this possibility by analyzing the overlap

of contralateral and ipsilateral projections in pups (P2-P12)”.

Line 348 presumably the Tukey's test followed a one-way ANOVA (otherwise it would

not be permitted with no significant interaction in the two-way ANOVA). This should be

clarified.

As we understand it, a 2-way ANOVA tests three null hypothesis at the same

time: 1) that the means at any level of independent variable 1 (age, in this case) are not

different, 2) that the means at any level of independent variable 2 (genotype, in this

case) are not different, and 3) that any effect of one independent variable does not

depend on the effect of the other variable. Thus, we found an effect of age, but not

genotype nor an interaction between the two and did a post-hoc test for differences

between all levels of age.
